# Chlamydial MreB Directs Cell Division and Peptidoglycan Synthesis in Escherichia coli in the Absence of FtsZ Activity

**DOI:** 10.1128/mBio.03222-19

**Published:** 2020-02-18

**Authors:** Dev K. Ranjit, George W. Liechti, Anthony T. Maurelli

**Affiliations:** a Emerging Pathogens Institute and Department of Environmental and Global Health, College of Public Health and Health Professions, University of Florida, Gainesville, Florida, USA; b Department of Microbiology and Immunology, Uniformed Services University of the Health Sciences, Bethesda, Maryland, USA; University of Queensland

**Keywords:** *Chlamydia*, MreB, RodZ, FtsZ, cell division, peptidoglycan, cell shape

## Abstract

Cell division is the ultimate process for the propagation of bacteria, and FtsZ is an essential protein used by nearly all bacteria for this function. *Chlamydiae* belong to a small group of bacteria that lack the universal cell division protein FtsZ but still divide by binary fission. Chlamydial MreB is a member of the shape-determining MreB/Mbl family of proteins responsible for rod shape morphology in Escherichia coli. *Chlamydia* also encodes a homolog of RodZ, an MreB assembly cytoskeletal protein that links MreB to cell wall synthesis proteins. We hypothesized that MreB directs cell division in *Chlamydia* and that chlamydial MreB could replace FtsZ function for cell division in E. coli. Overexpression of chlamydial *mreB-rodZ* in E. coli induced prominent morphological changes with production of large swollen or oval bacteria, eventually resulting in bacterial lysis. Low-level expression of chlamydial *mreB-rodZ* restored viability of a lethal Δ*mreB* mutation in E. coli, although the bacteria lost their typical rod shape and grew as rounded cells. When FtsZ activity was inhibited by overexpression of SulA in the Δ*mreB* mutant of E. coli complemented with chlamydial *mreB-rodZ*, spherical E. coli grew and divided. Localization studies using a fluorescent fusion chlamydial MreB protein indicated that chlamydial RodZ directs chlamydial MreB to the E. coli division septum. These results demonstrate that chlamydial MreB, in partnership with chlamydial RodZ, acts as a cell division protein. Our findings suggest that an *mreB-rodZ*-based mechanism allows *Chlamydia* to divide without the universal division protein FtsZ.

## Abstract (precis)

The study of *Chlamydia* growth and cell division is complicated by its obligate intracellular nature and biphasic lifestyle. *Chlamydia* also lacks the universal division protein FtsZ. We employed the cell division system of Escherichia coli as a surrogate to identify chlamydial cell division proteins. We demonstrate that chlamydial MreB, together with chlamydial RodZ, forms a cell division and growth complex that can replace FtsZ activity and support cell division in E. coli. Chlamydial RodZ plays a major role in directing chlamydial MreB localization to the cell division site. It is likely that the evolution of chlamydial MreB and RodZ to form a functional cell division complex allowed *Chlamydia* to dispense with its FtsZ-based cell division machinery during genome reduction. Thus, MreB-RodZ represents a possible mechanism for cell division in other bacteria lacking FtsZ.

## INTRODUCTION

The division of one bacterium into two bacteria is a deceptively simple process, and yet it involves one of the most complex molecular systems in bacterial physiology. Division begins with building a structure at midcell that comprises more than 20 dedicated proteins working together in a finely regulated manner to form a large complex known as the divisome (1, 2). Cell division in Escherichia coli and almost all other bacteria is orchestrated by FtsZ, a tubulin homologue protein that assembles into a ring structure (the Z-ring) at midcell in the cytoplasm and recruits at least 12 other essential Fts proteins and accessory proteins, including FtsA, ZipA, FtsE, FtsX, FtsL, FtsQ, FtsB, FtsW, and FtsN (3). The divisome directs synthesis of septal cell wall peptidoglycan, and cell wall hydrolases eventually facilitate the separation of two daughter cells (4, 5). Inactivation or depletion of FtsZ in E. coli prevents formation of the Z-ring with cessation of cell division, resulting in the production of filamentous cells (6, 7). Since FtsZ is conserved in a wide range of bacterial species, including mycoplasmas and archaea, it was thought that FtsZ plays a universal role in prokaryotic cell division (8–10). However, the members of *Chlamydiae* and *Planctomycetes* and Ureaplasma urealyticum are exceptions that lack FtsZ (11).

Present-day pathogenic chlamydiae separated from the last common ancestor that they shared with environmental chlamydiae roughly 700 million years ago (12). Their evolution to become successful obligate intracellular pathogens came by elimination of ancestral biosynthetic pathways and metabolic capabilities and subsequent strict reliance on the metabolism of their host cell for growth. This reductive process also had a spectacular impact on the cell division machinery. Whole-genome sequence analyses indicate that pathogenic chlamydiae disposed of all known FtsZ-driven cell division machinery proteins, including Z ring stabilization proteins FtsA, ZipA, and ZapA/B/C/D; septal localization proteins FtsE and FtsX; and recruiter proteins for cell wall synthesis FtsB and FtsP (13). The only FtsZ-driven cell division proteins retained in the pathogenic *Chlamydia* are chromosome segregation protein FtsK, lipid II flippase FtsW (a newly characterized peptidoglycan polymerase [14]), FtsQ, FtsL, and peptidoglycan synthase FtsI (PBP3) (13, 15). *Chlamydia* also contains a fully functional pathway for cell wall synthesis and MreB, an actin homologue that drives peptidoglycan synthesis on the side wall of most rod-shaped bacteria (16).

The function of MreB has been well characterized in E. coli (17, 18), Bacillus subtilis (19), Thermotoga maritima (20), and Caulobacter crescentus (21). Cylinder-shaped bacteria such as E. coli elongate along their longitudinal axis by using MreB and other proteins to form a complex called the elongasome (22, 23). MreB is a dynamic cytoskeletal protein that controls bacterial width by the spatiotemporal regulation of peptidoglycan synthesis on side walls. It is an essential molecule for cell elongation and survival. Depletion of MreB results in loss of rod shape, causing rounding or conversion into spherical cells (24, 25). MreB self-assembles into filamentous polymers, with discrete patches forming heterogenous puncta along the cell periphery. These MreB patches rotate around the long axis of the cell in a persistent manner, and this continuous rotation is required for cell wall synthesis (26). MreB rotation is mediated by the transmembrane protein RodZ, which couples MreB to the cell wall synthesis enzymes (27, 28). Assembly and localization of MreB are regulated by RodZ (29), and loss of RodZ leads to disassembly of MreB and consequent loss of rod shape (30).

The molecular mechanism of cell division in the *Chlamydia* genus in the absence of FtsZ is poorly understood. Two models proposed for *Chlamydia* cell division are canonical binary fission (31, 32) and polarized division (33). A subset of the members of *Planctomycetes* that lack FtsZ propagate by a budding-like mechanism (34). Cell division in the absence of FtsZ has been studied by inducing aberrant bodies or L-forms, a condition where the cell wall is lacking or lost. Atypical cell division in the L-form states of Listeria monocytogenes, Bacillus subtilis, E. coli, or Mycoplasma genitalium occurs in an erratic and random manner by membrane blebbing, budding, or stretching (35–38). *Chlamydia* also exhibits aberrant bodies under certain metabolic conditions that prevent cell division and generate metabolically active but nonreplicative forms (39). This state, known as persistence, is reversible, and under favorable conditions, aberrant bodies can resume normal cell division. In *Chlamydia*, MreB is implicated in cell division (40–42) but has not been extensively characterized. In this study, we examined the role of chlamydial MreB in cell division using E. coli as a surrogate system. We demonstrate that chlamydial MreB, with its cognate RodZ, enables the formation of a functional division complex in E. coli in the absence of FtsZ activity. Our results suggest that the resolution of the long-standing issue of how *Chlamydia* divides without the universal division protein FtsZ lies in the evolution of chlamydial MreB and RodZ to initiate cell division complex formation.

## RESULTS

### Overproduction of chlamydial MreB disrupts E. coli shape and cell division.

**FIG 1:**
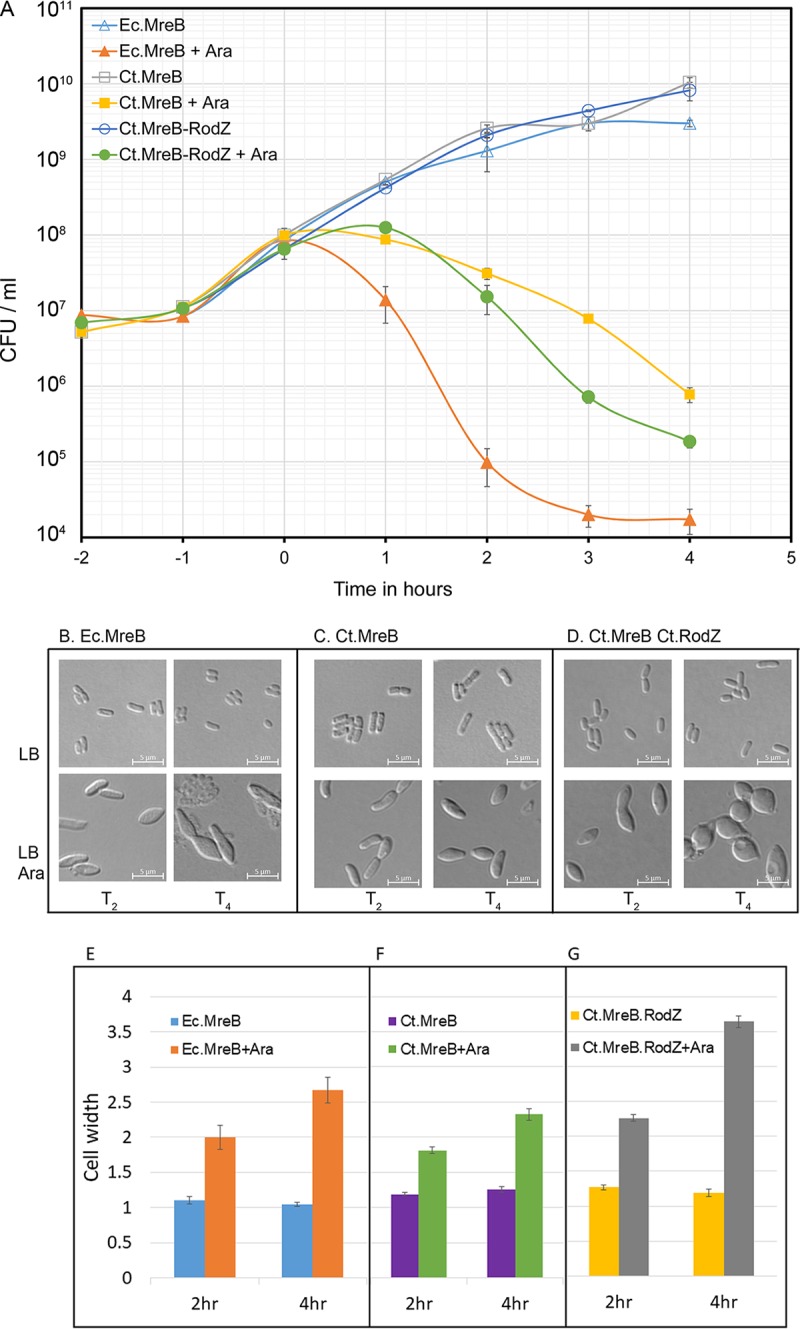
Overexpression of MreB disrupts E. coli growth and cell shape. (A) Exponentially growing cultures of E. coli MG1655 carrying *mreB*^Ec^, *mreB*^Ct^, or *mreB*^Ct^-*rodZ*^Ct^ in pBAD33 were induced with 0.2% arabinose (indicated as +Ara, with corresponding cultures remaining uninduced) at T_0_ (2 h after subculture from overnight), and viable counts were plotted against time. (B to G) Bacteria were examined for morphological changes (Ec.MreB [B], Ct.MreB [C], and Ct.MreB Ct.RodZ [D]) and cell width measurements (Ec.MreB ± Ara [E], EcCt.MreB ± Ara [G], and Ct.MreB Ct.RodZ ± Ara [G]) at 2 h and 4 h postinduction (T_2_ and T_4_). The images are representative of results from two independent experiments; *n* = 5.

As MreB determines cell shape and size in E. coli (16), we wanted to determine if chlamydial MreB (MreB^Ct^) has a similar functional role. We first examined the morphological effects of overproduction of E. coli MreB^Ec^ or chlamydial MreB^Ct^ on E. coli. The two genes were separately cloned into arabinose-inducible vector pBAD33. Since RodZ plays a critical role in MreB function (27) and chlamydial RodZ specifically interacts with chlamydial MreB (43), we constructed a transcriptional fusion of chlamydial MreB with chlamydial RodZ under the control of the arabinose promoter in pBAD33. E. coli cultures with these recombinant plasmids carrying MreB^Ec^, MreB^Ct^, or MreB^Ct^-RodZ^ct^ were induced by adding 0.2% arabinose. After induction, growth of E. coli cultures was significantly inhibited for all three constructs, with an accompanying decline in viable cell counts (Fig. 1A). MreB^Ec^ overproduction resulted in the most pronounced growth inhibitory effect, whereas overexpression of MreB^Ct^-RodZ^ct^ produced greater inhibition than overexpression of MreB^Ct^ alone (Fig. 1A). As expected, results of morphological examinations performed at 2 h and 4 h postinduction indicated a gradual increase in midcell diameter resulting in swollen or oval bacteria (Fig. 1B to G). Interestingly, overexpression of MreB^Ec^ and MreB^Ct^ produced identical changes in morphology, with midcell diameter increasing by about 2-fold (Fig. 1E and F), whereas MreB^Ct^-RodZ^ct^ converted E. coli rods into prominent spherical or oval shapes with >3-fold increases in midcell diameter (Fig. 1D and G). The increase in midcell diameter indicates that these cells fail to elongate (44), possibly due to interruption in side wall peptidoglycan synthesis. It is likely that the higher levels of MreB competing for cell wall synthesis proteins disrupted the normal cell wall building process and that chlamydial MreB behaves similarly to E. coli MreB under these conditions. Moreover, the presence of chlamydial RodZ further enhanced the effect of chlamydial MreB on cell diameter and growth inhibition, suggesting that chlamydial MreB functions are coupled to its cognate RodZ. Thus, these experiments indicate that overexpression of chlamydial MreB can result in cell growth and morphology phenotypes identical to those seen with E. coli MreB.

### Chlamydial MreB partially complements E. coli MreB.

**FIG 2:**
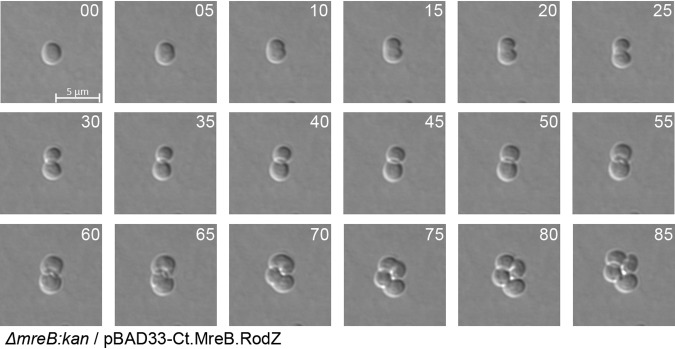
Cell division in the complemented strains. Exponentially growing cultures were placed on LB soft agar pads on microscope slides, and growth was recorded by time-lapse microscopy (time in minutes displayed in top right corner). The images are representative of results from two independent experiments.

Amino acid alignment of MreB from Chlamydia trachomatis as well as other organisms, including Bacillus subtilis, E. coli, Helicobacter pylori, and Treponema pallidum, indicates that the chlamydial homolog contains conserved structural regions essential for the function of eukaryotic actin (45). These similarities suggest that chlamydial MreB also is a functional bacterial cytoskeletal protein. Since overproduction of chlamydial MreB exhibited phenotypes functionally similar to those seen with E. coli MreB overproduction, we next asked if chlamydial MreB could complement an E. coli *mreB* deletion mutant. We utilized P1 transduction and a plasmid carrying *sdiA* (a suppressor of *mreB* knockout lethality) to transfer *mreB^Ec^*::*kan* from donor strain SKMG14-1 (44) and generated an *mreB* deletion in E. coli carrying pBAD33-Ct.MreB and in E. coli carrying pBAD33-CtMreB.RodZ (see Materials and Methods). Transductants in both genetic backgrounds lost the chromosomal copy of *mreB^Ec^* and grew under conditions of basal (i.e., no induction) expression of pBAD33-CtMreB or pBAD33-CtMreB.RodZ. While both MreB^Ct^ and MreB^Ct^-RodZ^Ct^ supported growth of the *mreB^Ec^* deletion mutants, E. coli lost its rod shape and grew as spherical cells (see Movie S1 in the supplemental material). Time-lapse microscopy of individual cells revealed that each spherical cell developed a one-sided notch by 5 to 10 min which deepened to split the cells by 20 to 30 min (Fig. 2, time zero to 30 min). Surprisingly, cell division and growth phenotypes were similar whether strains were complemented by MreB^Ct^ or by MreB^Ct^-RodZ^Ct^ (data not shown). The control *mreB* knockout strain complemented with E. coli *mreB* in *trans* (Δ*mreB*:*kan*/pBAD33-Ec.MreB) grew as regular rods under conditions of basal expression (no induction) of pBAD33-EcMreB (data not shown).

10.1128/mBio.03222-19.6 MOVIE S1 Exponentially growing cultures were placed on LB soft agar pads on microscope slides, and growth (in minutes) was recorded (top left corner) by time-lapse microscopy for E. coli Δ*mreB*:kan/pBAD33-Ct.MreB-RodZ. Download Movie S1, AVI file, 7.4 MB. Copyright © 2020 Ranjit et al. 2020 Ranjit et al. This content is distributed under the terms of the Creative Commons Attribution 4.0 International license.

To determine if chlamydial MreB can direct peptidoglycan synthesis in growing spherical cells, we utilized the fluorescent d-alanine derivative HADA (hydroxy-coumarin-carbonyl-amino-d-alanine) to label peptidoglycan (46). Spherical cells took up HADA and labeled the peptidoglycan layer (see Fig. S1B and C in the supplemental material), indicating active peptidoglycan synthesis as seen with the rod-shaped E. coli control (Fig. S1A). These results suggest that chlamydial MreB can support peptidoglycan synthesis in an E. coli mutant lacking *mreB*. We did not observe any additional complementation phenotypes with expression of chlamydial RodZ. The E. coli Δ*mreB* mutant still contains a functional FtsZ protein, and it is probable that FtsZ builds the septal wall for cell division whereas chlamydial MreB supports peptidoglycan synthesis around the spheres.

10.1128/mBio.03222-19.1 FIG S1 Chlamydial MreB directs peptidoglycan synthesis in E. coli. For peptidoglycan labeling, fluorescent d-alanine (HADA) was added to the growing culture for 1 h and the results were imaged under fluorescent microscopy; (A) E. coli Δ*mreB*:*kan*/pBAD33-Ec.MreB. (B) E. coli Δ*mreB*:*kan*/pBAD33-Ct.MreB-RodZ. (C) E. coli Δ*mreB*:*kan*/pBAD33-Ct.MreB-RodZ. Download FIG S1, PDF file, 0.3 MB. Copyright © 2020 Ranjit et al. 2020 Ranjit et al. This content is distributed under the terms of the Creative Commons Attribution 4.0 International license.

### Chlamydial MreB drives cell division in E. coli when FtsZ is inactivated but requires chlamydial RodZ.

**FIG 3:**
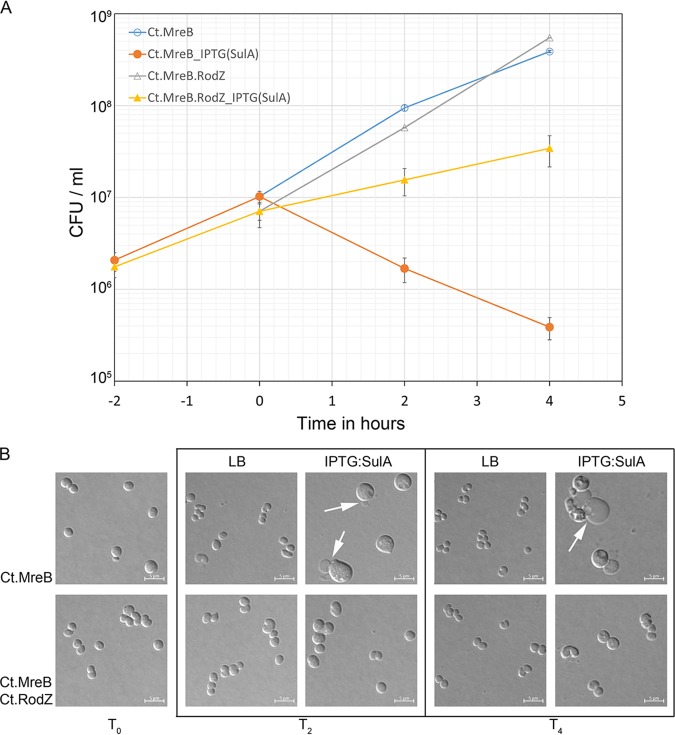
Chlamydial MreB and RodZ rescue FtsZ inhibition in E. coli. (A) Exponentially growing cultures of Δ*mreB^Ec^* E. coli carrying pBAD33-Ct.MreB or pBAD33-Ct.MreB-RodZ along with pSulA were induced with 100 μM IPTG T_0_ (2 h after subculture from overnight), and viable counts were plotted against time. (B) Samples taken before induction and 2 h and 4 h postinduction (T_0_, T_2_, and T_4_) were examined by light microscopy for morphological changes. Arrows indicate blebs. Images are representative of results from two independent experiments.

Since *Chlamydia* grows and divides without FtsZ, we wanted to test the hypothesis that chlamydial MreB can support cell division in E. coli in the absence of FtsZ activity. To examine this possibility, we used SulA to inhibit FtsZ polymerization in the complemented mutant strains. In E. coli, SulA inhibits cell division by blocking the GTPase activity of FtsZ, preventing FtsZ polymerization and resulting in cell filamentation (6, 47). First, we examined the ability of a plasmid carrying *sulA* under the control of an IPTG (isopropyl-β-d-thiogalactopyranoside)-inducible promoter to inhibit cell division and induce filamentation in wild-type E. coli. When induced with 100 μM IPTG, *sulA* effectively inhibited cell division in wild-type E. coli producing filamentous cells (Fig. S2A and B). To verify the efficacy of SulA in blocking FtsZ polymerization at the division site in the presence of chlamydial genes in E. coli, we induced SulA in the presence of pBAD33-ctMreB.RodZ. Under these conditions, the cells formed filaments (Fig. S2C). When the *sulA* plasmid was transformed into the Δ*mreB* mutant carrying *mreB^Ct^* in *trans* and induced with 100 μM IPTG, growth was inhibited, and viable counts continued to decline through 4 h postinduction whereas the uninduced culture continued to grow (Fig. 3A). However, when the *sulA* plasmid was transformed into and induced in the Δ*mreB* mutant carrying *mreB^Ct^-rodZ^Ct^*, the culture continued to grow, and the viable cell count continued to increase although at a lower rate than in the uninduced culture (Fig. 3A).

10.1128/mBio.03222-19.2 FIG S2 Effect of SulA induction on wild-type E. coli. (A) Exponentially growing cultures of E. coli MG1655 carrying IPTG-inducible pSulA were induced with 100 μM IPTG at T_0_ (2 h of growth), and viable counts were plotted against time. (B) E. coli MG1655/pSulA. (C) E. coli MG1655/pBAD33-Ct.MreB-RodZ/pSulA. At time points T_0_ (inducer added), T_2_, and T_4_, cells were examined by light microscopy for morphological changes. Download FIG S2, PDF file, 0.3 MB. Copyright © 2020 Ranjit et al. 2020 Ranjit et al. This content is distributed under the terms of the Creative Commons Attribution 4.0 International license.

Examined by light microscopy, the uninduced spherical *mreB^Ct^* strain was seen to have maintained a spherical shape whereas, after *sulA* induction, these spherical cells started to change in shape and size, converting into enlarged spheroids (Fig. 3B). These enlarged spheroids started to show blebs indicating possible inner-content leakage (Fig. 3B, at T_2_ and T_4_ arrow) and these spheroids developed vesicles. The defects in shape and size and the decline in viable counts suggested that the presence of chlamydial MreB alone failed to rescue cell division under FtsZ-inactivating conditions. However, the morphology (shape and size) of chlamydial MreB-RodZ spheres seen when SulA was induced to inhibit FtsZ remained unaffected (Fig. 3B lower panel). The increase in the viable counts of the chlamydial MreB-RodZ culture suggests that chlamydial RodZ is needed to facilitate chlamydial MreB-directed growth and cell division in E. coli when FtsZ is inactivated. These observations further suggest that chlamydial MreB does not utilize E. coli RodZ to support cell division in E. coli but requires its cognate RodZ.

**FIG 4:**
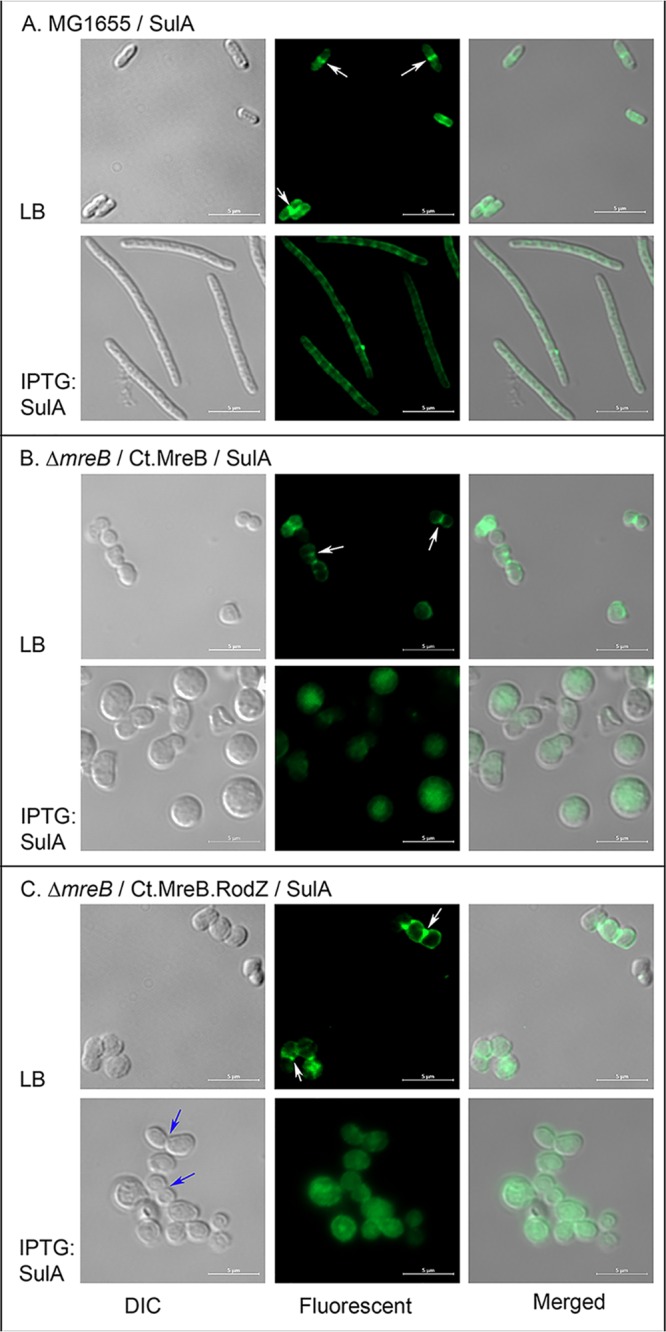
Immunolabeling of FtsZ during *sulA* induction. *sulA* was induced by addition of 100 μM IPTG for 2 h to inhibit FtsZ polymerization at the division site in cells carrying a copy of *sulA* in *trans* and immunolabeled to detect FtsZ. E. coli MG1655/pSulAz (A), Δ*mreB*:*kan*/pBAD33-Ct.MreB/pSulA (B), and Δ*mreB*:*kan*/pBAD33-Ct.MreB-RodZ/pSulA (C) were grown under uninduced (LB) and induced (IPTG:SulA) conditions. White arrows indicate FtsZ septum formation, and blue arrows indicate visible cell division. Images are representative of results from two independent experiments. DIC, differential interference contrast.

To ensure that FtsZ polymerization was effectively inhibited by *sulA* induction in our parent and mutant E. coli strains, we utilized immunostaining of FtsZ to detect septal FtsZ localization under *sulA* induction conditions. When *sulA* was induced in *trans* for 2 h by addition of 100 μM IPTG, the filamentous cells in wild-type E. coli failed to show a complete FtsZ septation band compared to cells without *sulA* induction (Fig. 4A, LB [Luria-Bertani] for uninduced and IPTG:SulA for induced). We then examined a Δ*mreB* mutant strain carrying *mreB^Ct^* and *sulA* in *trans*. Under uninduced conditions, FtsZ was detected as punctate foci at one side of the cell, possibly aiding in cell division (Fig. 4B, LB, white arrows). However, under *sulA* induction conditions, FtsZ labeling became diffuse, indicating that FtsZ was unable to form a division plane (Fig. 4B, IPTG:SulA). Similarly, in the case of the Δ*mreB* mutant carrying *mreB^Ct^-rodZ^Ct^*, under uninduced conditions, FtsZ appeared as punctate foci at one side of the dividing cells, indicating some FtsZ polymerization to support cell division (Fig. 4C, LB, white arrows). Interestingly, under conditions of *sulA* induction on these spherical Δ*mreB* cells carrying *mreB^Ct^-rodZ^Ct^*, FtsZ exhibited diffuse cytoplasmic staining, indicating that SulA was able to depolymerize FtsZ. These observations confirm that SulA is effective at inhibiting FtsZ polymerization at the division site under conditions of induction in *mreB* mutant strains carrying either *mreB^Ct^* or *mreB^Ct^-rodZ^Ct^* in *trans*. This result supports our conclusion that the MreB^Ct^-RodZ^Ct^ complex is capable of driving division in E. coli in the absence of FtsZ polymerization and localization to the division plane.

PBP3 (FtsI) is the major peptidoglycan transpeptidase enzyme involved in bacterial cell division and is part of the division complex in E. coli (48). Inhibition of PBP3 by cephalexin induces filamentation (49, 50). To determine if chlamydial MreB/RodZ-dependent division in E. coli requires the activity of this peptidoglycan synthase, we utilized cephalexin, an inhibitor of PBP3. We examined the morphology of E. coli cells 2 h after treatment with 50 μg/ml cephalexin, which produced long filaments in MG1655 (Fig. S3). Similar cephalexin treatment of the *mreB* knockout strain carrying Ct.MreB.RodZ and SulA in *trans* produced large, nondividing spherical bodies in both the presence and absence of SulA induction (Fig. S3). These observations indicated that the chlamydial MreB-RodZ cell division process in E. coli is dependent on peptidoglycan synthesis and requires the E. coli peptidoglycan synthase PBP3.

10.1128/mBio.03222-19.3 FIG S3 Effect of cephalexin on morphology of E. coli. Exponentially growing cultures of E. coli MG1655 (left panel) and Δ*mreB*:*kan*/pBAD33-MreB-RodZ/pSulA (middle and right panels) were treated with 50 μg/ml cephalexin (lower panel) for 2 h. SulA was left uninduced (no IPTG) (middle panel) or induced with 100 μM IPTG (right panel). Download FIG S3, PDF file, 0.3 MB. Copyright © 2020 Ranjit et al. 2020 Ranjit et al. This content is distributed under the terms of the Creative Commons Attribution 4.0 International license.

### Chlamydial RodZ directs MreB localization to the division septum in E. coli.

**FIG 5:**
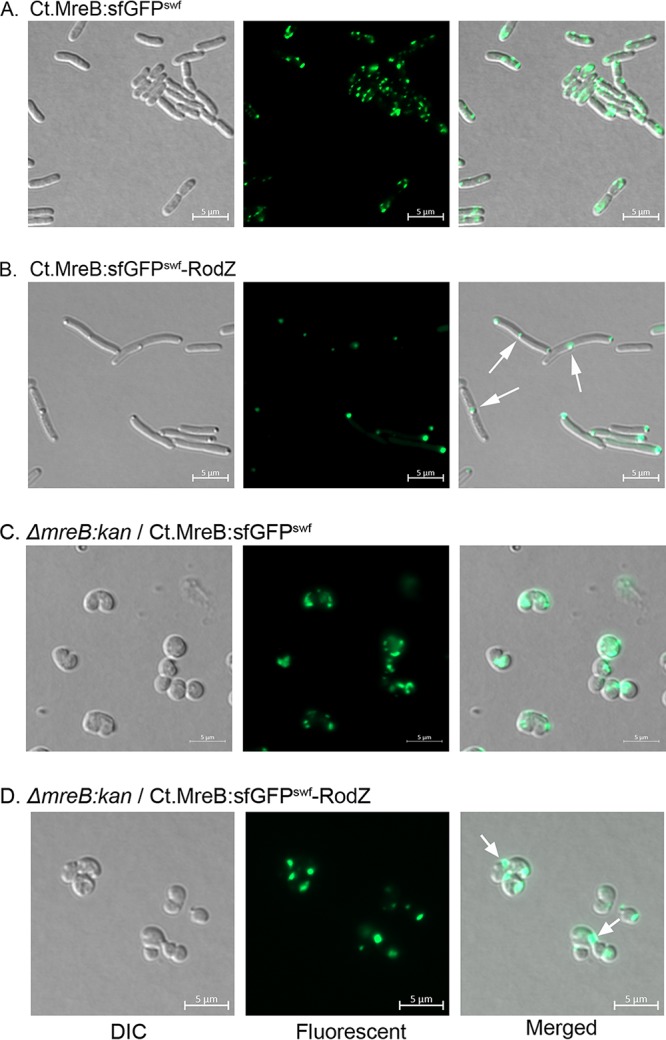
Chlamydial RodZ directs chlamydial MreB to the division site. Exponentially growing cultures of E. coli strains carrying a sfGFP sandwich fusion in chlamydial MreB were induced with 0.02% arabinose and grown for 1 h before imaging by fluorescence microscopy. (A) E. coli/pBAD33-Ct.MreB:sGFP^swf^. (B) E. coli pBAD33-Ct.MreB:sGFP^swf^-RodZ. (C) Δ*mreB*:*kan*/pBAD33-Ct.MreB:sGFP^swf^. (D) Δ*mreB*:*kan*/pBAD33-Ct.MreB:sGFP^swf^-RodZ. Arrows indicate division septum. Images are representative of results from two independent experiments.

As chlamydial MreB appears to drive cell division in E. coli, it needs to localize to the cell division site. We hypothesize that chlamydial RodZ plays a role in directing MreB localization to this site. To examine this possibility, we designed a fluorescent fusion derivative of chlamydial MreB to monitor its localization in E. coli. We generated a sandwich fusion in chlamydial MreB (Ct.MreB:sGFP^swf^) by inserting superfolder green fluorescent protein (sfGFP) (30, 51) between positions G247 and G248 of chlamydial MreB (see Table S2 in the supplemental material) and linked its expression to the arabinose-inducible promoter in pBAD33. A similar sandwich fusion approach was used previously to study the function of E. coli MreB (30, 51). Additionally, to study the role of chlamydial RodZ, we constructed a transcriptional fusion of the sandwich fusion *mreB* and the native chlamydial *rodZ*, resulting in Ct.MreB:sGFP^swf^-RodZ. To confirm that these constructs were functional, we looked for morphological defects produced by overexpression in E. coli. At high levels of induction (0.2% arabinose), both the pBAD33-Ct.MreB:sGFP^swf^ and pBAD33-Ct.MreB:sGFP^swf^-RodZ sandwich constructs produced morphological defects similar to those seen with the corresponding wild-type genes (data not shown). However, when the level of induction was low (0.02% arabinose), we detected the fluorescence signals without any prominent morphological defects in E. coli. At 1 h postinduction, E. coli cells carrying pBAD33-Ct.MreB:sGFP^swf^ showed fluorescent signals in multiple foci dispersed throughout the side wall of the cells (Fig. 5A). Surprisingly, when we examined E. coli cells carrying pBAD33-Ct.MreB:sGFP^swf^-RodZ, fluorescent chlamydial MreB produced single foci localized to the division plane or in the polar position (Fig. 5B, arrows). These results indicate that cellular localization of chlamydial MreB in E. coli is determined by its cognate RodZ. Thus, chlamydial RodZ directs chlamydial MreB toward the division septum, which goes on to become a set of polar regions after completion of cell division.

To further analyze the functional role and localization pattern of these sandwich fusions, we transferred these constructs into the E. coli Δ*mreB* mutant. Δ*mreB* mutants carrying the sandwich fusion pBAD33-Ct.MreB:sGFPswf or pBAD33-Ct.MreB:sGFP^swf^-RodZ were viable and spherical, indicating that sandwich fusions were able to partially complement the Δ*mreB* mutation in E. coli. When the sandwich fusion was subjected to a low level of induction (0.02% arabinose), the chlamydial MreB signal was detected 1 h postinduction. As shown in Fig. 5C and D, strains expressing Ct.MreB:sGFP^swf^ showed fluorescent signals in multiple foci and patches dispersed around the sphere whereas the presence of chlamydial RodZ in the strains (Ct.MreB:sGFP^swf^-RodZ) resulted in the production of fluorescent signals localized only at the division site (arrow in Fig. 5C and D). These observations further support the model that chlamydial RodZ directs MreB to the division site in E. coli.

## DISCUSSION

The molecular mechanism of bacterial cell division and growth has been well characterized in E. coli, providing a useful surrogate system to examine the function of divergent or heterologous division proteins (52). Because of the obligate intracellular nature of *Chlamydia*, we utilized E. coli to examine the functional role of chlamydial MreB in cell division. The shape defects generated by overproduction of chlamydial MreB in E. coli imply interactions with one or more E. coli shape-determining proteins. Such interactions can generate competition between chlamydial MreB and E. coli MreB and prevent the proper assembly of the E. coli MreB complex to regulate shape and size. Bacteria such as E. coli that grow in size by elongation utilize several proteins for establishing, maintaining, and regulating cell shape. The actin homologue MreB plays a key role among other proteins to form an elongasome (22, 23). Wachi and Matsuhashi previously demonstrated that supplying additional copies of *mreB* results in elongated shapes in E. coli (53), indicating that a critical balance of all participating shape-determining proteins is required for formation of a stable elongasome that maintains a defined cellular shape. Our observation of shape defects in E. coli under conditions of E. coli MreB overproduction supports the idea that excess MreB competing for other elongasome proteins disrupts the proper assembly of the elongasome. In this context, our observations indicate that chlamydial MreB disrupts the elongasome when overproduced and suggest that it is a functional protein capable of interacting with E. coli shape-determining proteins. In the E. coli MreB complex, one of the best-characterized relationships is that between MreB and RodZ (27). In our overexpression studies, we observed dramatic shape defects when overproduction of chlamydial MreB was coupled with chlamydial RodZ. We surmise that the chlamydial MreB-RodZ complex competes with the E. coli MreB-RodZ complex to provoke extensive disruption of the E. coli elongasome. Thus, chlamydial MreB and RodZ exhibited the properties of shape-determining proteins.

Shape defects generated by disruption of the elongasome are attributed to disruption of MreB-dependent peptidoglycan synthesis. MreB is also a negative regulator of cell division (53), possibly via disruption of the division complex. In our overproduction studies, both E. coli MreB and chlamydial MreB not only inhibited cell growth but also led to cell lysis. Lethality caused by shape-related proteins has also been reported previously in Bacillus subtilis by overproducing an MreB isoform (54) and in Helicobacter pylori by overproducing MreC (23). Similarly, disruption of an endogenous cytoskeleton system by a heterologous MreB protein in B. subtilis, where expression of *mreB* from Clostridium perfringens produced shape defects and cell lethality, has been reported previously (55). These results suggest that heterologous shape-determining proteins influence elongasome stability. Although blockage of cell division initiation by mislocalization of the peptidoglycan synthesis machinery is an alternative explanation (23), we believe that disruption of the MreB complex activates peptidoglycan hydrolases that damage the peptidoglycan layer, causing cell death. Nevertheless, in this context, chlamydial MreB appears fully functional; it interrupts the E. coli elongasome when overproduced, and it causes lethality similar to that caused by overproduction of E. coli MreB.

Although our overproduction studies suggested that chlamydial MreB is functional in E. coli, there is no direct molecular basis for the conclusion that chlamydial MreB is a shape-determining protein. Depletion of MreB in E. coli causes cells to lose their rod shape and become spheres which eventually enlarge and lyse (44). Although a deletion of *mreB* in E. coli is lethal, suppression of the *mreB* defect occurs with multicopy expression of *ftsQAZ* or SdiA induction of the promoter upstream of *ftsQAZ* (*ftsQ2p*) (44). However, whereas cell division is restored in these suppressor mutants, rod shape is lost and the bacteria divide as spheres. Similarly, chlamydial MreB was able to suppress the lethality of an *mreB* deletion in E. coli but could not restore the rod shape. We propose that chlamydial MreB suppresses the lethality of an *mreB* deletion without restoring the rod shape by acting either as a growth-supporting protein or as a division protein. The shape-determining regions of E. coli MreB have been mapped (16, 56). Although mutations in these regions cause loss of rod shape, cells maintain growth and remain viable. Therefore, the first possibility is that chlamydial MreB behaves as an altered E. coli MreB that keeps a Δ*mreB* mutant viable by maintaining growth. In this case, chlamydial MreB is a growth-supporting protein. As for the second possibility, overproduction of FtsZ in E. coli suppresses the lethality of a *mreB* mutation but the cells lose their rod shape and grow as spheres (44). We propose that, in an analogous fashion, chlamydial MreB behaves as an FtsZ (i.e., a division protein) to suppress the lethality of the *mreB* knockout. Thus, chlamydial MreB is a division protein. These two mechanisms are not mutually exclusive, and it is possible that chlamydial MreB is both a growth-supporting protein and a division protein.

To test the model of chlamydial MreB acting as a division protein, we inactivated FtsZ in our *mreB* mutant strains. Failure of chlamydial MreB alone to support E. coli growth would suggest that the chlamydial MreB is not a division protein. However, when chlamydial RodZ was combined with chlamydial MreB, growth of E. coli was sustained even when FtsZ was inactivated. In *Chlamydia*-related species, RodZ is recruited to the division septum before MreB (57), indicating that RodZ plays a leading role in recruiting MreB to the division site. Our studies with the superfolder GFP fusion to chlamydial MreB demonstrated that localization of chlamydial MreB is dependent on chlamydial RodZ and that it directs chlamydial MreB to the cell division site in E. coli. These observations provide a novel paradigm for bacterial cell division in an organism lacking FtsZ; i.e., chlamydial MreB together with its cognate RodZ can replace the activity of the essential cell division protein FtsZ in the divisome of E. coli and support cell division.

Although structurally distinct, MreB, a key component of the elongasome, and FtsZ, a key component of the divisome, might be functionally similar because both direct peptidoglycan synthesis, albeit at different cellular locations. The elongasome is thought to have evolved as a modified version of the divisome (22). It is possible that MreB is less constrained to polymerize around any inner surface of the cell as it requires fewer interacting partners than the more stringent FtsZ. If that is true, MreB acts as a growth-supporting protein when localized on the side wall and behaves as a cell division protein when localized at the division plane. In the case of chlamydial RodZ, we propose that it functions like a guide protein to recruit chlamydial MreB to the division plane in *Chlamydia* and to fulfil the role of a division protein in E. coli.

Taking the data together, chlamydial MreB is functional in the E. coli cell division system and localizes to the division plane in the presence of chlamydial RodZ. More importantly, chlamydial MreB suppresses the lethality of a Δ*mreB* mutation in E. coli by behaving like a growth protein by itself or by behaving like a cell division protein when expressed together with chlamydial RodZ. Therefore, we propose that chlamydial MreB is a functional cell growth and cell division protein.

## MATERIALS AND METHODS

### Bacterial strains, plasmids, DNA manipulation, and media.

The bacterial strains and plasmids used in the study are listed in Table 1. The primers and oligonucleotides used to create each plasmid are listed in Table S1 in the supplemental material. Bacteria were grown in Luria-Bertani (LB) medium; when appropriate, kanamycin (50 μg/ml), chloramphenicol (20 μg/ml), spectinomycin (50 μg/ml), or cephalexin (50 μg/ml) was added. Standard DNA, PCR, and molecular biological techniques were utilized for cloning and plasmid construction (58), and E. coli DH5α was used as the intermediate cloning strain. All experiments were performed in the E. coli MG1655 background. For chlamydial genomic DNA, Chlamydia trachomatis serovar L2 strain 434/Bu (kindly provided by H. Caldwell, Rocky Mountain Laboratory) was used. The correctness of each plasmid was verified by DNA sequencing.

**TABLE 1:** Strains and plasmids used in this study

Strain or plasmid	Relevant genotype	Source and/or reference
E. coli strains		
MG1655	F^−^ λ*^−^ ilvG rfb-50 rph-1*	62
DH5α	Φ80Δ*lacZ*ΔM15 Δ(*lacZYA-argF*)*U169 deoR recA1 endA hsdR17*(*r*_k_^−^ *m*_k_^+^) *phoA supE44 thi-1gyrA96 relA1*	63
ATM1499	MG1655/pBAD33-Ct.MreB	This work
ATM1501	MG1655/pBAD33-Ec.MreB	This work
ATM1503	MG1655/pBAD33-Ct.MreB-RodZ	This work
SKMG14-1	MG1655 Δ*mreB*::kan/pCX16	Kevin Young (44)
ATM1506	MG1655/pTS-sdiA	
ATM1507	MG1655 Δ*mreB*::kan/pBAD33-Ec.MreB	This work
ATM1508	MG1655 Δ*mreB*::kan/pBAD33-Ct.MreB	This work
ATM1509	MG1655 Δ*mreB*::kan/pBAD33-Ct.MreB-RodZ	This work
ATM1512	MG1655/pSulA	This work
ATM1513	MG1655 Δ*mreB*::kan/pBAD33-Ct.MreB/pSulA	This work
ATM1514	MG1655 Δ*mreB*::kan/pBAD33-Ct.MreB-RodZ/pSulA	This work
ATM1517	MG1655/pBAD33-Ct.MreB:sGFP^swf^	This work
ATM1518	MG1655/pBAD33-Ct.MreB:sGFP^swf^-RodZ	This work
ATM1519	MG1655 Δ*mreB*::kan/pBAD33-Ct.MreB:sGFP^swf^	This work
ATM1520	MG1655 Δ*mreB*::kan/pBAD33-Ct.MreB:sGFP^swf^-RodZ	This work
ATM1540	MG1655/pBAD33-Ct.MreB-RodZ/pSulA	This work
Plasmids		
pBAD33	pACYC184 *cat araC P_araB_*	64
pBAD33-Ct.MreB	pBAD33-*mreB^Ct^*	This work
pBAD33-Ec.MreB	pBAD33-*mreB^Ec^*	This work
pBAD33-Ct.MreB-RodZ	pBAD33-*mreB^Ct^ rodZ^Ct^*	This work
pKD46	*repA101*(ts) *oriR101 bla P_araB_-γ β exo*	65
pTS-sdiA	*repA101*(ts) *oriR101 bla P_araB_-sdiA*	This work
pSulA	*colE1 lacI^q^ aadA P_lac_-sulA*	This work
pBAD33-Ct.MreB:sGFPswf	pBAD33*-mreB^ct^*::*sfgfp*::*mreB^ct^*	This work
pBAD33-Ct.MreB:sGFPswf-RodZ	pBAD33-*mreB^ct^*::*sfgfp*::*mreB^ct^ rodZ^ct^*	This work

10.1128/mBio.03222-19.4 TABLE S1 Primer list. Download Table S1, PDF file, 0.4 MB. Copyright © 2020 Ranjit et al. 2020 Ranjit et al. This content is distributed under the terms of the Creative Commons Attribution 4.0 International license.

### Plasmid construction.

Plasmids were constructed in pBAD33 using primers listed in Table S1. Genomic DNA from E. coli MG1655 was used to clone E. coli genes (*mreB* and *sulA*), whereas genomic DNA of C. trachomatis L2 was used to clone chlamydial genes (*mreB^Ct^*, *rodZ^Ct^*). Genes were amplified by PCR and cloned using restriction sites (underlined in the primer sequences). To create the superfolder GFP sandwich fusions in chlamydial MreB, sequences of sfGFP were inserted at G2478 and D248 of chlamydial MreB (Table S2) as follows. First, sfGFP was amplified by adding *mreB^ct^* homology overhangs on flanking regions by using primers DP354 and DP355 (Table S1B). Next, two *mreB^ct^* fragments amplified by using primers DP123 and DP352 (5′ fragment) and primers DP124 and DP353 (3′ fragment). The three fragments were combined at equimolar ratios and fused together by using splicing by overlap extension as described previously (59), and the product was cloned into pBAD33. To construct *sdiA*, a temperature-sensitive plasmid was constructed by modifying pKD46 as follows. First, the segment containing the multiple-cloning site from pBAD33 was PCR amplified by using primers DP117 and DP185 and digested with SacI and NcoI. Next, pKD46 was digested with SacI and NcoI to remove the segment containing the λ red genes. The backbone segment of pDK46 (without λ red genes) was gel extracted and ligated with the segment containing the multiple-cloning site from pBAD33, generating a temperature-sensitive plasmid, pTS-BAD. To clone *sdiA* into pTS-BAD, *sdiA* was PCR amplified from E. coli MG1655 by using primers DP187 and DP188. The PCR product was digested with SacI and SalI and cloned into pTS-BAD to generate pTS-*sdiA*.

10.1128/mBio.03222-19.5 TABLE S2 Sandwich fusion scheme. Download Table S2, DOCX file, 0.02 MB. Copyright © 2020 Ranjit et al. 2020 Ranjit et al. This content is distributed under the terms of the Creative Commons Attribution 4.0 International license.

### Strain construction.

To construct the Δ*mreB* mutant in E. coli MG1655, first, E. coli MG1655 was transformed with pTS-*sdiA*, and then a P1 lysate was prepared using donor strain SKMG14-1 (kindly provided by Kevin Young). Transductions into recipient strains of MG1655/pTS-*sdiA* carrying pBAD33-Ec.MreB, pBAD33-Ct.MreB, pBAD33-Ct.MreB-RodZ, pBAD33-Ct.MreB:sGFP^swf^, or pBAD33-Ct.MreB:sGFP^swf^-RodZ were carried out using this P1 lysate, and the strains were grown at 30°C. Transductants were screened for the Δ*mreB*::*kan* deletion by using primers DP183 and DP184 for flanking regions of *mreB*. To cure pTS-*sdiA*, each recipient strain was grown at 42°C and subsequently screened for ampicillin-sensitive colonies.

### Time-lapse microscopy and peptidoglycan labeling.

For time-lapse microscopy growth observation, microscope slides were covered with LB soft agar (0.7% agar) and the cells were placed on the surface of the soft agar pad. Slides were placed onto the stage of a Zeiss Axio Image.Z1 microscope with an enclosed incubation chamber at 37°C. To label peptidoglycan, growing cultures were mixed with 500 μM fluorescent d-alanine derivative hydroxy-coumarin-carbonyl-amino-d-alanine (HADA), a gift from Michael S. VanNieuwenhze. Cells were further incubated for 1 h, washed twice with phosphate-buffered saline (PBS), and prepared for microscopy. To detect fluorescence, DAPI (4′,6-diamidino-2-phenylindole) filters (358-nm excitation and 461-nm emission wavelengths) were used.

### FtsZ immunofluorescent staining.

Immunostaining of FtsZ was performed as described previously (60) with some modifications (61). Briefly, E. coli cells were fixed in 2.6% (vol/vol) paraformaldehyde and 0.04% (vol/vol) glutaraldehyde for 15 min at room temperature and then incubated on ice for 20 min. After fixation, the cells were resuspended in PBS with the addition of NaBH_4_ (1 mg/ml) for 5 min followed by three washings in PBS and resuspension in GTE buffer (50 mM glucose, 10 mM EDTA, 20 mM Tris-HCl, pH 7.5). Cells were then immobilized on coverslips coated with poly-l-lysine by allowing cells to settle on the glass surface for 10 min. Cells were permeabilized with 0.1% Triton X-100–PBS for 5 min and then treated with lysozyme at 10 μg/ml for 5 min followed by two washes in PBS. Next, bovine serum albumin (BSA) solution (20 mg/ml in PBS) was added for 30 min to block nonspecific antibody binding. After that, cells were incubated with primary rabbit anti-FtsZ antibody (Agrisera, catalogue no. AS10715) diluted 1:200 in BSA solution (20 mg/ml in PBS) at 4°C overnight. The following day, samples were washed thoroughly with 0.01% Tween 20–PBS. Next, the cells were incubated with secondary antibody, goat anti-rabbit IgG conjugated with Alexa Fluor Plus 488 (Thermo Fisher, catalogue no. A32731) diluted 1:100 in BSA solution (20 mg/ml in PBS) at room temperature for 1 h. The cells were thoroughly washed with Tween 20 solution before imaging was performed on a Zeiss Axio Image.Z1 microscope.
