# Supplementary figures and images for: Chlamydial MreB Directs Cell Division and Peptidoglycan Synthesis in Escherichia coli in the Absence of FtsZ Activity
Source: mBio. 2020 Feb 18;11(1):e03222-19. doi: 10.1128/mBio.03222-19 (PMC7029139; doi:10.1128/mBio.03222-19)

A. Ec.MreB

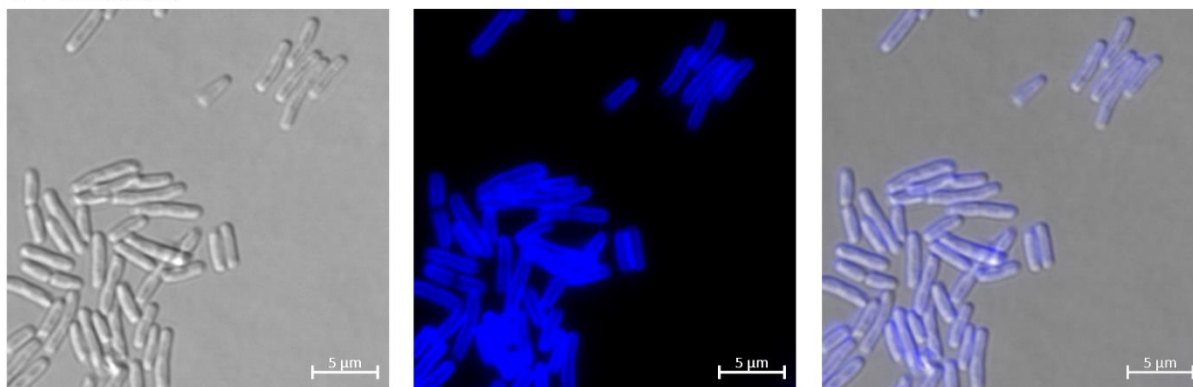

B. Ct.MreB

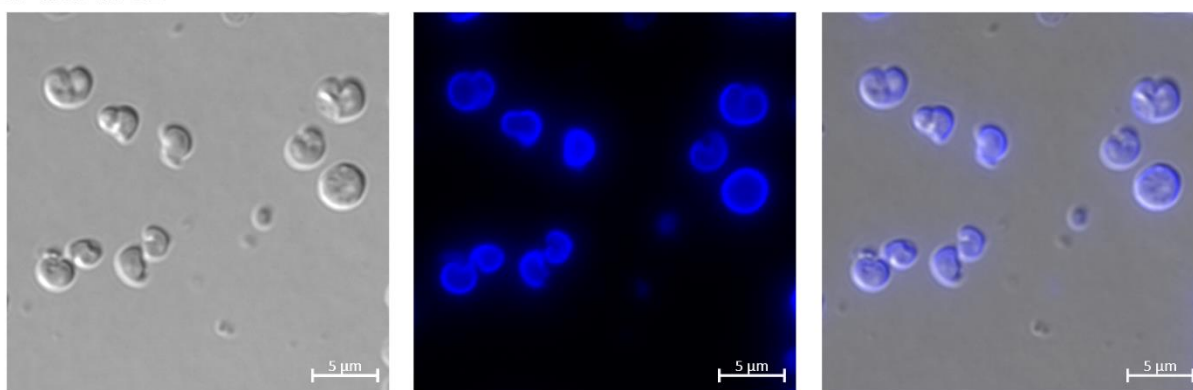

C. Ct.MreB-RodZ

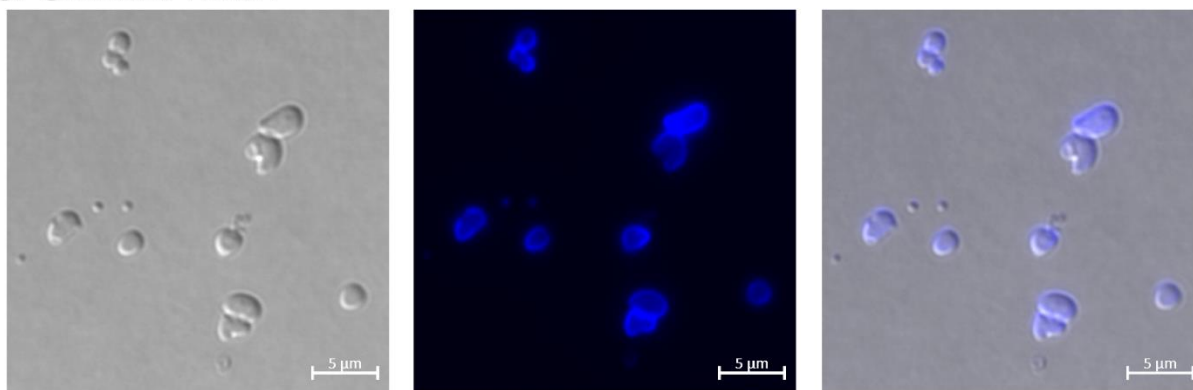

DIC

HADA

Merge

FIG S1

Supplement: FIG S1 [file mBio.03222-19-sf001.pdf]

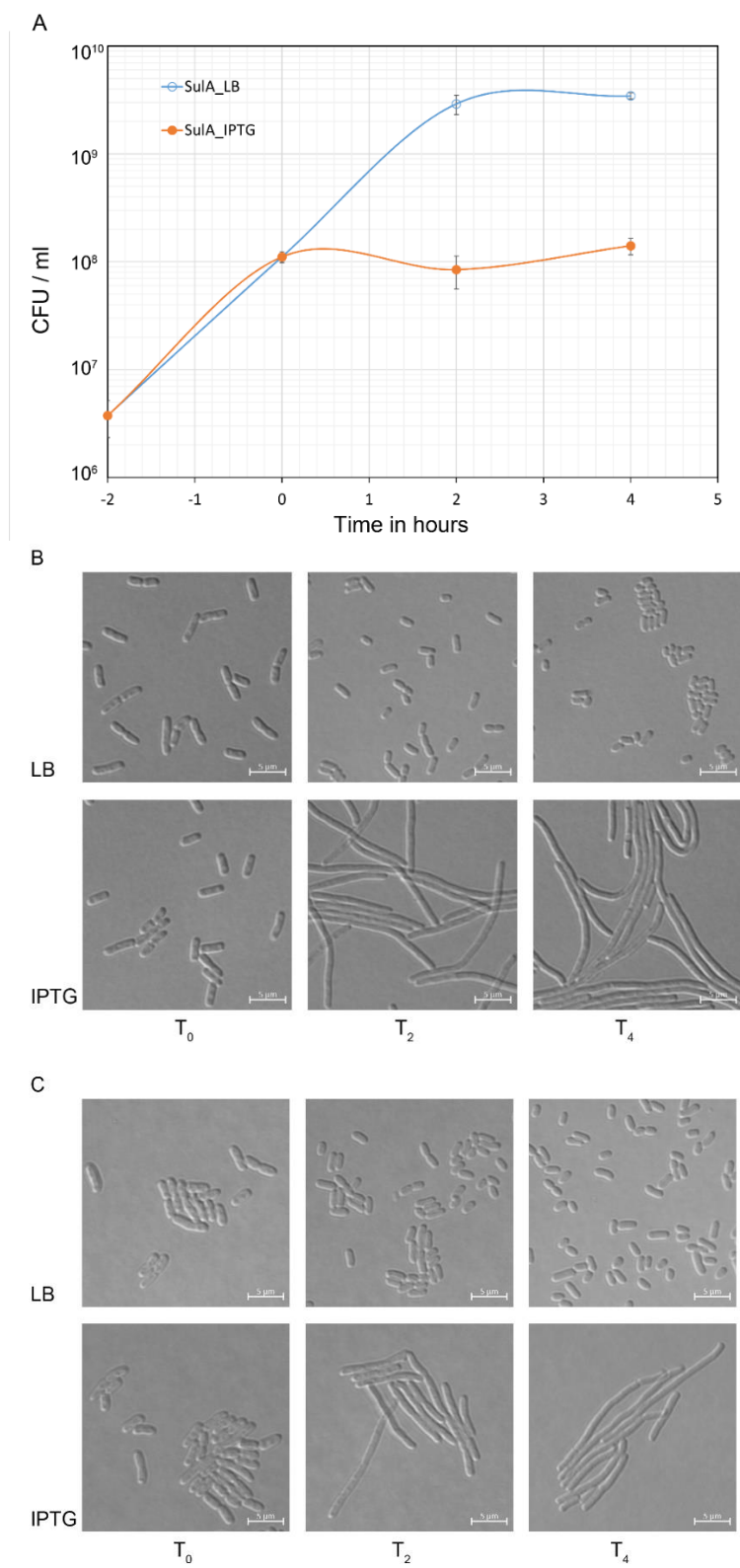

FIG S2

Supplement: FIG S2 [file mBio.03222-19-sf002.pdf]

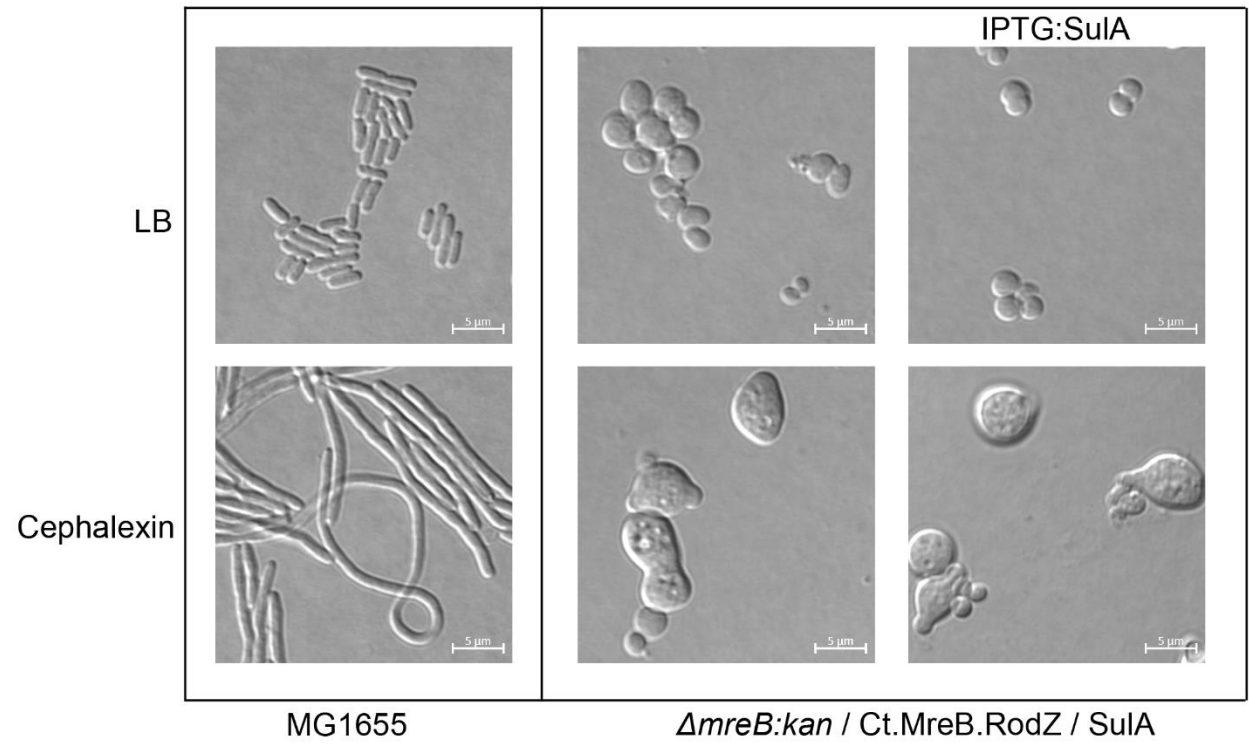

FIG S3

Supplement: FIG S3 [file mBio.03222-19-sf003.pdf]
