# Supplementary material for: Chlamydial MreB Directs Cell Division and Peptidoglycan Synthesis in Escherichia coli in the Absence of FtsZ Activity
Source: mBio. 2020 Feb 18;11(1):e03222-19. doi: 10.1128/mBio.03222-19 (PMC7029139; doi:10.1128/mBio.03222-19)
Supplement: TABLE S1 [file mBio.03222-19-st001.pdf]

SUPPORTING MATERIALS

Table S1 Primer List

Primer Name	Used to construct	Sequences
DP123	pBAD33-Ct.MreB	cgcGGTACCCTGATTAACTTTATAAGGAGGAAAAACATATGAGCCCATACCGCAGC
DP124	pBAD33-Ct.MreB	cgcGTCGACTTATACTAAACTCTCTTTTCGTT
DP181	pBAD33-Ec.MreB	cgcGAGCTCCTGATTAACTTTATAAGGAGGAAAAACATATGTTGAAAAAATTTCGTGGCA
DP182	pBAD33-Ec.MreB	cgcGTCGACTTACTCTTCGCTGAACAGGT
DP371	pBAD33-Ct.MreB-RodZ	TTTAGTATAAGTCGACGATTAACTTTATAAGGAGGAAAAACATATGAGCGAACATGTCCACA
DP372	pBAD33-Ct.MreB-RodZ	ATGCCTGCAGGTCGACCTAGAAAAGGTTGAATAGATTCCCT
DP354	pBAD33-Ct.MreB:sGFP^{swf}	AGGTTCTGCTTATCCGTTAGGTAGCGGCAGCAGCTCTAAAGGTGAAGAACTGTTCA
DP355	pBAD33-Ct.MreB:sGFP^{swf}	TCCATCTCCAATTCCTGATCGCCCAGCGGCGCGCCGCTTTTGTAGAGCTCATCCATGCCG
DP352	pBAD33-Ct.MreB:sGFP^{swf}	AGGTTCTGCTTATCCGTTAGGT
DP353	pBAD33-Ct.MreB:sGFP^{swf}	TCCATCTCCAATTCCTGATC
