# Supplementary material for: Chlamydial MreB Directs Cell Division and Peptidoglycan Synthesis in Escherichia coli in the Absence of FtsZ Activity
Source: mBio. 2020 Feb 18;11(1):e03222-19. doi: 10.1128/mBio.03222-19 (PMC7029139; doi:10.1128/mBio.03222-19)
Supplement: TABLE S2 [file mBio.03222-19-st002.docx]

Table S2. Sandwich fusion scheme: The amino acid sequences of chlamydial MreB showing insert region for sandwich fusion construction. Green highlighted region (XXX) indicates the sequence of monomeric super-folder green fluorescence (sfGFP). Linker amino acid sequences are highlighted in grey.

MSPYRSLYKIKHLSNRLYNKALGRFDRVFNFFSGNVGIDLGTANTLVYVRGRGIVLSEPSVVAVDAQTHAVLAVGHKAKAMLGKTPRKIVAVRPMKDGVIADFEIAEGMLKALIKRVTPARSMFRPKILIAVPSGITGVEKRAVEDSALHAGAQEVILIEEPMAAAIGVDLPVHEPAASMIIDIGGGTTEIAIISLGGIVESRSLRIAGDEFDECIINYMRRTYNLMIGPRTAEEIKITIGSAYPLGSGSSXXXSGAPLGDQELEMEVRGRDQVAGLPITKRINSVEIRECLAEPIQQIIECVRLTLEKCPPELSADLVERGMVLAGGGALIKGLDKALSKNTGLSVITAPHPLLAVCLGTGKALEHLDQLKKRKESLV

Ct.MreB

sfGFP

SKGEELFTGVVPILVELDGDVNGHKFSVRGEGEGDATNGKLTLKFICTTGKLPVPWPTLVTTLTYGVQCFSRYPDHMKQHDFFKSAMPEGYVQERTISFKDDGTYKTRAEVKFEGDTLVNRIELKGIDFKEDGNILGHKLEYNFNSHNVYITADKQKNGIKANFKIRHNVEDGSVQLADHYQQNTPIGDGPVLLPDNHYLSTQSVLSKDPNEKRDHMVLLEFVTAAGITHGMDELYK
